# Seropositivity and Risk Factors for *Toxoplasma gondii* and *Neospora caninum* in Intensive Dairy Cattle from Different Farms in Central Chile

**DOI:** 10.3390/ani16101456

**Published:** 2026-05-09

**Authors:** Catalina Godoy-Alfaro, Camila Muñoz-Zanzi, Sofía Jara-Méndez, Catalina Tapia, Mario Duchens, Carlos Núñez, Camila Varela, Raúl Alegría-Morán, Patricio Retamal, Galia Ramírez-Toloza

**Affiliations:** 1 Laboratory of Parasitology and Parasitic Diseases, Department of Animal Preventive Medicine, Faculty of Veterinary and Livestock Sciences, Universidad de Chile, Santiago 8820808, Chile; cata.godoy.a@gmail.com (C.G.-A.); camilamunozzanzi@gmail.com (C.M.-Z.); sofia.jara.m@ug.uchile.cl (S.J.-M.); catalina.tapia.b@ug.uchile.cl (C.T.); 2 Faculty of Veterinary Medicine and Agronomy, Universidad de las Américas, La Florida-Campus, Santiago 8250122, Chile; 3 Department of Animal Science, Faculty of Veterinary and Livestock Sciences, Universidad de Chile, Santiago 8820808, Chile; mduchens@uchile.cl; 4 Department of Clinical Sciences, Faculty of Veterinary and Livestock Sciences, Universidad de Chile, Santiago 8820808, Chile; canunez@uchile.cl; 5 Laboratory of Infectious Diseases, Department of Animal Preventive Medicine, Faculty of Veterinary and Livestock Sciences, Universidad de Chile, Santiago 8820808, Chile; camila.varela@ug.uchile.cl (C.V.); pretamal@uchile.cl (P.R.); 6 Escuela de Medicina Veterinaria, Facultad de Medicina, Facultad de Ciencias Biológicas y Facultad de Agronomía y Sistemas Naturales, Pontificia Universidad Católica de Chile, Santiago 7820436, Chile; raul.alegria@uc.cl

**Keywords:** dairy cattle, *Toxoplasma gondii*, *Neospora caninum*, seroprevalence, risk factors, BCG

## Abstract

*Toxoplasma gondii* and *Neospora caninum* are apicomplexan parasites infecting cattle, with implications for public health and livestock productivity, respectively. Since effective vaccines against these parasites are not currently available, identifying epidemiological factors associated with infection is important for improving control strategies. This study aimed to estimate the seroprevalence of both parasites and to identify factors associated with seropositivity in intensive dairy cattle in central Chile. A cross-sectional study was conducted using serum samples from 567 cattle, analyzed by ELISA. Epidemiological data were collected through semi-structured surveys, and associations with seropositivity were evaluated using multivariable logistic regression models, including mixed-effects models to account for farm-level clustering. Seroprevalence was 7.6% for *T. gondii* and 22.4% for *N. caninum*. For *T. gondii*, factors associated with seropositivity included older age categories (OR = 7.09; 11.25) and the presence of dogs in pens (OR = 6.07). For *N. caninum*, straw bedding use (OR = 5.13) and cat presence (OR = 6.32) were associated with higher odds of seropositivity. An additional association with lower *N. caninum* seropositivity was observed for BCG vaccination (OR = 0.24). These findings provide updated epidemiological data for dairy cattle in Chile. The association observed with BCG vaccination should be interpreted cautiously, as the study design does not permit causal inference.

## Simple Summary

*Toxoplasma gondii* and *Neospora caninum* are parasites that infect cattle and may affect both public health and animal production. Because effective vaccines against these parasites remain unavailable, identifying epidemiological factors associated with infection is important for improving control strategies. In this study, we analyzed 567 serum samples from dairy cattle to estimate the prevalence of these infections and identify factors associated with seropositivity. The prevalence was 7.6% for *T. gondii* and 22.4% for *N. caninum*. Several farm-management factors, including bedding type and the presence of dogs or cats, were associated with seropositivity. These findings contribute to the understanding of the epidemiology of these parasites in dairy cattle and highlight management-related factors that may influence infection dynamics in livestock production systems. An additional association with *N. caninum* seropositivity was observed for BCG vaccination, although this result requires further evaluation in specifically designed studies.

## 1. Introduction

Protozoan parasites belonging to the phylum Apicomplexa cause economic losses and animal health problems in cattle worldwide [1,2]. *Toxoplasma gondii* and *Neospora caninum* are apicomplexan parasites that infect cattle and share notable similarities in their life cycles and routes of infection; however, they differ in their sanitary effects. While *T. gondii* has significant public health implications [3], *N. caninum* primarily causes reproductive losses in livestock [4].

Toxoplasmosis is a zoonotic disease recognized as the fourth most significant foodborne illness worldwide [5]. Felines are the definitive hosts of *T. gondii*, which release oocysts into the environment through feces, contaminating water and food for humans and animals [6]. In intermediate hosts, such as livestock, the parasite begins an extra-enteric cycle, forming tissue cysts mainly in nervous, ocular, skeletal, and cardiac muscles, which can infect other animal species [7]. In many species, including humans, *T. gondii* can be transmitted congenitally, causing abortion, reproductive and neurological issues, and symptoms in immunosuppressed individuals [7,8]. Since it is estimated that between one-third and one-half of the global population is infected with *T. gondii*, it is considered a disease of great importance. Nonetheless, its prevalence varies significantly among countries, regions, and animal species [8]. 

*Neospora caninum* is not a zoonotic disease; instead, it is a pathogen that affects cattle and canids [9]. Domestic and wild canids serve as definitive hosts, with infection mainly occurring when they ingest intermediate hosts containing tissue cysts [10], thereby completing the intestinal cycle. Oocysts are shed in feces and contaminate water and food sources. After sporulation, these parasitic forms can be ingested by cattle. When a pregnant cow ingests the parasite, tachyzoites can reach the placenta, leading to abortion or the birth of an infected calf. This process, known as transplacental exogenous transmission, is linked to abortion storms [11]. There is also endogenous transplacental transmission, which occurs in congenitally infected females, in which the infection is reactivated during pregnancy [11]. Therefore, *N. caninum* is considered a significant cause of economic and productive losses in the dairy industry [4,12,13].

The control strategies mainly focus on implementing farm biosecurity protocols, hygiene measures, and management practices to lower oocyst levels in the environment [14]. Biosafety practices include keeping cats and dogs away from ruminant areas, food storage zones, and water supplies, as well as removing placenta and fecal material and conducting plague control, all of which have been identified as risk factors in some studies [14]. Despite the relevance of both forms of parasitosis in cattle, there is a critical shortage of data on the risk factors driving positivity in Chile and worldwide. Current information is largely limited to prevalence reports, leaving key variables—such as interaction dynamics with definitive hosts (felines and canines), the sanitary quality of water sources, and vaccine use—unidentified.

Although vaccination is seen as a promising and cost-effective way to prevent transmission of both agents, its efficacy and safety remain limited [14,15,16]. For *T. gondii*, there are vaccines to prevent shedding in cats or to reduce tissue cyst development and abortions [15]. However, these vaccines are live, have a limited shelf life, and pose a risk of infection to humans who handle them [15]. Regarding *N. caninum*, a commercially available killed vaccine with variable efficacy for pregnant cows was available until 2009 [14].

In this context, *Mycobacterium bovis* Bacillus Calmette-Guérin (BCG), a vaccine used to protect against tuberculosis, provides non-specific immunity against other infectious diseases and certain cancers [17]. This homologous and heterologous immune protection is primarily mediated by trained immunity, which involves the development of immunological memory in innate immune cells following initial exposure to an infection or vaccination [18]. The protection induced by BCG has been tested against several infectious agents, including *Candida albicans* [19], *Legionella pneumophila* [20], *Streptococcus pneumoniae* [21], and H1N1 influenza virus [22]. However, there is limited information about its effect on protozoan parasites. There are reports about its impact on *Babesia* spp. [23,24] and *Plasmodium* spp. [24,25] in mice and on *Leishmania major* [26] in humans. However, the effect of this vaccine on the prevalence of *T. gondii* and *N. caninum* has not been tested.

In Chile, a limited number of farms in the Central zone have experimentally incorporated this vaccine and tested its effects on *M. tuberculosis* prevalence and on productive parameters [27,28]. Thus, it would be interesting to investigate its potential role in modifying the prevalence of these parasitic diseases.

In summary, despite the global importance of *T. gondii* and *N. caninum* infections in cattle, epidemiological information from intensive dairy production systems in Chile remains limited. Based on these considerations, the aim of this study was to assess the prevalence and identify risk factors associated with protozoa in dairy cattle in central Chile to inform more effective control strategies.

## 2. Materials and Methods

A cross-sectional observational study was conducted between March 2023 and July 2024. Animals were selected through convenience sampling from 14 intensive dairy production systems located in the central zone of Chile, including farms in the Valparaíso Region (V; *n* = 3), the Metropolitana Region (MR; *n* = 9), and the O’Higgins Region (VI; *n* = 2). The sampling strategy was based on voluntary farm participation and logistical accessibility. For each sampled animal, blood samples were collected, and epidemiological information was recorded through semi-structured surveys administered at the farm level. These surveys collected data related to herd management practices and environmental conditions, which were later used for epidemiological characterization and risk factor analysis.

### 2.1. Sample Size Calculation

The sample size was calculated using sampling equations for determining proportions or means [29]. For this study, a 95% confidence level and a 5% margin of error were used.

The estimated dairy cattle population for Regions V, RM, and VI is 21,306 heads [30]. Given the limited knowledge of *T. gondii* prevalence in cattle in the study area, the sample size was calculated assuming a prevalence of 50%, ensuring the minimum possible sample size [29]. Regarding *N. caninum*, a prevalence of 22.43% (83/370) was found in dairy herds in the Southern Zone of Chile [31]. A 95% confidence level and a 5% precision were also set. Cattle samples were selected based on information from the agricultural census conducted by Instituto Nacional de Estadística [32].

Based on these data, the minimum corrected sample sizes were 377 for *T. gondii* and 264 for *N. caninum*; however, a total of 567 samples were collected. This total was used to estimate the seroprevalence for both parasites. The study included females of any productive or reproductive status from a dairy farm with intensive production management, weighing more than 250 kg, as estimated using Quetelet’s formula.

A total of 14 farms were included in the study. Within each farm, the proportion of sampled animals was estimated relative to the total herd size and ranged from 1% to 47%, depending on herd size. Five of the 14 farms recruited in this study were enrolled in a BCG vaccination program. Animals in this program were immunized with BCG between the first days of life and 14 months of age, with a mean age at vaccination of 6.15 months. Blood samples used in the present study were collected between 7 and 28 months after vaccination, with a mean interval of 14.6 months between BCG administration and sampling.

### 2.2. Sample Collection

Blood samples were collected by venipuncture of the coccygeal vein into 10 mL sample tubes without anticoagulant and transported to the Laboratory of Parasitology and Parasitic Diseases, Faculty of Veterinary and Livestock Sciences, Universidad de Chile. Upon arrival, the serum was isolated into 1.5 mL Eppendorf tubes and centrifuged at 3000 rpm for 10 min using a BOECO^®^ U-320R centrifuge (BOECO, Hamburg, Germany). Each serum was transferred to a new 1.5 mL Eppendorf tube and stored at −20 °C until analysis.

### 2.3. Immunoassay by Enzyme-Linked Immunosorbent Assay (ELISA)

Serum antibodies to *Toxoplasma gondii* and *N. caninum* were detected using two commercially available indirect ELISA kits. To detect immunoglobulin G anti-*T. gondii* antibodies, an ID Screen^®^ Toxoplasmosis Indirect Multi-species ELISA kit (IDVet, Innovative Diagnostics, Grabels, France) was used according to the manufacturer’s instructions. The specificity for ruminants is 99.42% (95% CI: 98.8–100%), and sensitivity is 98.36% (95% CI: 95.29–99.44%). Briefly, negative and positive controls and samples were diluted 1:10 and added to microplate wells. The microplates were incubated for 45 min at 21 °C. Afterward, the wells were washed with the wash solution, and a conjugated secondary antibody was added. The microplates were incubated for 30 min at 21 °C. The wells were then washed, and the microplates were incubated with the revealed solution for 15 min at 21 °C. Finally, a stop solution was added, and the microplates were measured at 450 nm in an ELISA reader plate (BioTek ELx800, BioTek Instruments, Inc., Winooski, VT, USA) to determine optical density (OD). To interpret the results, an S/P% was calculated using the following formula:

$$S/P\%=\frac{{OD}_{ample}-{OD}_{negative\;control}}{{OD}_{positive\;control}-{OD}_{negative\;control}}\times 100$$

Results greater than or equal to 50% were considered positive; results less than 40% were considered negative; results greater than or equal to 40% and less than 50% were considered doubtful. Samples considered doubtful were retested. A second doubtful result was considered a negative sample.

To detect immunoglobulin G anti-*N. caninum*, an ID Screen^®^ *Neospora caninum* Competition kit (IDVet, Innovative Diagnostics, Grabels, France) was used according to the manufacturer’s instructions. This kit has 100% specificity (95% CI: 99.63–100%) and 100% sensitivity (95% CI: 98.8–100%) in cattle. Briefly, 50 µL from each sample was added to each well and incubated for 45 min at 37 °C in a humid chamber. The microplate was washed three times, and 100 µL of an anti-*N. caninum* conjugate antibody was added to each well. Samples were incubated at 5 °C for 30 min, then washed three times. After that, 100 µL of substrate solution was added to each well, incubated at 5 °C for 15 min, and finally, 100 µL of stop solution was added. The OD was measured at 450 nm using a plate reader (BioTek ELx800). The results were calculated as the percentage S/N (S/N%), using the following formula:

$$S/N\%=\frac{{OD}_{sample}}{{OD}_{negative\;control}}\times 100$$

Results of 50% or less were considered positive; more than 60% were considered negative; and if the result was greater than 50% and less than or equal to 60%, it was considered doubtful. Doubtful samples were retested. A second doubtful result was considered a negative sample.

### 2.4. Epidemiological Survey

Epidemiological information was collected from different farms using two surveys, focusing on relevant parasite information, and, in addition, specific information was requested from the sampled cattle. The required animal and farm information was primarily drawn from previous studies on *T. gondii* and *N. caninum* in cattle, as well as on the risk factors associated with these infections. These include information on the presence and access of dogs, cats, and rodents; water sources and animal feed; age; number of calvings and abortions; and cattle access to grazing or browsing areas [12,33,34,35,36,37,38,39,40,41,42,43,44,45]. Additionally, the materials used for animal bedding and for drinking and feeding troughs, as well as their locations on farms, were examined. Once the database was established, certain variables were excluded from the study due to insufficient variability across farms, including the presence of rodents and their control, and inconsistencies in information on reproductive problems on most farms. In addition, the number of animals included in the risk factor analysis was reduced because four of the farms did not fully complete the epidemiological survey; consequently, 458 animals (10 farms) were used instead of 567 (14 farms).

### 2.5. Statistical Analysis

Univariable logistic regression analysis (*p* < 0.15) was performed on all potential risk factors reported in the survey. Then, Fisher’s exact test or Chi-square test was used to assess collinearity and associations between variables. Subsequently, individual-level data were used to fit conventional multivariable logistic regression models using a backward stepwise elimination procedure based on the Likelihood Ratio Test (LRT). Variables that were not significant in model comparison (*p* > 0.05) were removed from the model. Variables that were not statistically significant during model construction, but whose removal changed the regression coefficients of the remaining variables by more than 20%, were retained in the final model to adjust for potential confounding.

To evaluate whether farm-level clustering materially influenced the results, multivariable mixed-effects logistic regression models were also fitted by including farm as a random intercept. For these models, univariable mixed-effects logistic regression analyses (*p* < 0.15) were first conducted as a screening step, and the selected variables were subsequently included in the multivariable mixed-effects models. The contribution of the random effect to model variability was assessed by estimating the farm-level variance and the intraclass correlation coefficient (ICC). The conventional multivariable model was used as an initial reference analysis, whereas the final interpretation was based on the mixed-effects model when the random effect was estimable and non-negligible. When the mixed-effects model was singular, or the random-effect variance was estimated as zero, the conventional multivariable model was considered the more parsimonious and interpretable approach. The convergence criterion for the non-mixed models was set to epsilon (ε) = e^−16^, whereas mixed-effects models were fitted using the bobyqa optimizer with an increased maximum number of function evaluations to ensure stable convergence. Biologically and epidemiologically plausible interactions were also evaluated. All analyses were performed using R (version 4.5.1) and RStudio (version 2025.05.1+513).

### 2.6. Biosafety and Bioethics Certificates

To carry out the handling and intervention on the animals, as well as the subsequent processing and manipulation of the samples, this study was approved by the Institutional Committee for the Care and Use of Animals (23666-VET-UCH) and the Committee of Biosafety (Certificate No. 191), Universidad de Chile.

## 3. Results

**Figure 1:**
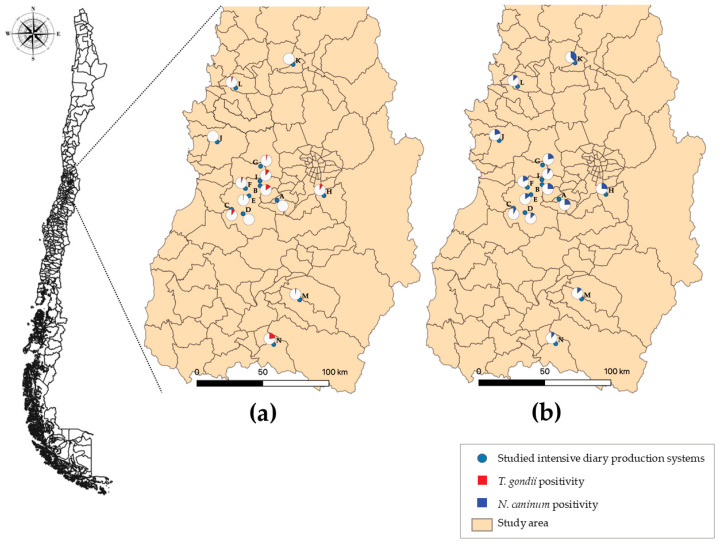
Spatial distribution of the farms under study (A–N) and seropositivity map for *T. gondii* (**a**) and *N. caninum* (**b**). The corresponding colors within the white circles indicate the proportions of cattle seropositive for each parasite, among the total number of cows sampled on the farm.

Five hundred and sixty-seven serum samples were collected from 14 farms distributed throughout the Central Zone of Chile. Nine farms were located in the MR, three in the V Region, and two in the VI Region (Figure 1). The prevalence of *T. gondii* was 7.6% (43/567), and 22.4% (127/567) for *N. caninum* (Table 1). Regarding coinfection, only 7 animals were seropositive for both parasites.

To assess the potential impact of excluding farms with incomplete epidemiological information, the full dataset (*n* = 567) was compared with the restricted dataset (*n* = 458) that included only farms with complete epidemiological data. Differences in proportions, 95% confidence intervals, and null hypothesis testing assuming a difference equal to 0 were calculated using a chi-square approximation, following Rothman [46]. No statistically significant differences were observed between datasets for *T. gondii* seroprevalence (7.58% vs. 8.73%; *p*-value = 0.578; 95% CI: −4.7 to 2.4 percentage points), *N. caninum* seroprevalence (22.34% vs. 22.49%; *p*-value = 1.000; 95% CI: −5.3 to 5.1 percentage points), or the proportion of BCG-vaccinated animals (49.21% vs. 52.84%; *p*-value = 0.248; 95% CI: −9.8 to 2.5 percentage points). These comparisons indicate that excluding farms with incomplete epidemiological information did not materially alter the main descriptive estimates.

The results of the univariable logistic regression screening used to construct the conventional multivariable model for *T. gondii* are presented in Supplementary Table S1, and the corresponding conventional multivariable logistic regression model is presented in Supplementary Table S2. In the mixed-effects logistic regression model for *T. gondii*, the farm-level random effect was estimable and non-negligible (variance = 0.828; SD = 0.910), and the model did not show singularity; the adjusted ICC was 0.201, and the marginal and conditional R^2^ values were 0.226 and 0.381, respectively. The statistically significant variables associated with *T. gondii* seropositivity in the multivariable mixed-effects logistic regression model and identified as risk factors were age 3 to 4.5 years (OR = 11.25), age greater than 4.5 years (OR = 7.09), and presence of dogs in the pens (OR = 6.07) (Table 2).

In the mixed-effects logistic regression model for *N. caninum*, the farm-level random-effects variance was estimated as zero, and the model was singular. Consequently, the ICC and the conditional R^2^ could not be estimated, whereas the marginal R^2^ was 0.159. This indicates that, after accounting for the fixed effects, no relevant residual clustering remained at the farm level. Therefore, in accordance with the analytical strategy described in the Materials and Methods, the conventional multivariable logistic regression model was retained as the most parsimonious and interpretable model for this outcome. The results of the univariable logistic regression screening used to construct the conventional multivariable model for *N. caninum* are presented in Supplementary Table S3. Among the statistically significant variables associated with *N. caninum* seropositivity in the multivariable logistic regression model, the following were identified as risk factors: the use of straw bedding (OR = 5.13) and the presence of cats (OR = 6.32). The variables associated with lower odds of seropositivity were: the entry of animals into the shed (OR = 0.02), the use of BCG (OR = 0.24), and the presence of dogs belonging to the property (OR = 0.12), as well as the presence of dogs from the property and unknown dogs (OR = 0.16) (Table 3).

## 4. Discussion

*Neospora caninum* and *T. gondii* are intracellular coccidian parasites with a worldwide distribution [47]. *N. caninum* is an important cause of abortion in cattle [48]. Although *T. gondii* does not play a significant role in reproductive disorders in cattle [49], this species remains a source of foodborne toxoplasmosis in humans [47], being a public health concern. In this study, the seroprevalence of *T. gondii* in cattle was 7.6%. This seroprevalence is lower than the global estimate (16.9%) [50] and lower than that reported for dairy cattle in Brazil (31.4%) [51], but similar to the 7.9% reported in Holstein cattle in Japan [52]. It is likely that the main factors influencing these differences were farm management practices and climatic conditions. The latter was evident in this study, where *T. gondii* seropositivity increased as the sampling area moved southward (0% in the V region, 6% in the MR, and 19% in the VI region). This gradient effect has been previously identified in other locations, including Mongolia [53], where higher seroprevalence rates were recorded in the Central regions than in the Western region, suggesting that geographical location may be a risk factor for toxoplasmosis. This effect could be explained, in part, by the temperature. A study conducted by Gomes et al. [42] found that an average annual temperature (AAT) between 28.5 and 31.1 °C is a risk factor for *T. gondii* infection in livestock, compared with higher AATs. In Chile, the sampled regions differ in AAT and proximity to the sea, so it seems reasonable to investigate whether these differences affect the occurrence of toxoplasmosis in cattle. Although this result is supported by the literature, it is necessary to verify whether differences in sample sizes across selected regions influenced the results, which constitutes a limitation of this study.

For *T. gondii*, the mixed-effects model indicated that farm-level variability contributed to the explanation of seropositivity, and the final interpretation was therefore based on this model. The significant risk factors retained were the age range of 3 to 4.5 years (OR = 11.25), age over 4.5 years (OR = 7.09), and the presence of dogs in the pens (OR = 6.07).

As reported previously, this study found that risk factors for *T. gondii* infection in cattle include older age [44,45], which is associated with greater exposure to the agent. However, in this case, the highest risk was observed in cattle aged 3 to 4.5 years rather than in those older than 4.5 years. This could be due to a higher number of samples in the 3 < x ≤ 4.5 years range (99) than in the >4.5 years range (59). Another risk factor found was the presence of dogs in the pens. This may be due to dogs rolling in feces and consuming feces of other animals, in this case, cats, which can spread oocysts in their fur or through their feces [54,55]. It is also important to consider that the presence of dogs in the pens could indicate shortcomings in the farms’ infrastructure and biosecurity protocols.

The seroprevalence of *N. caninum* was 22.4%, slightly higher than the overall prevalence of 20% reported by Ribeiro et al. [56] in 2019, and lower than the 24% reported in South America. In Chile, the seropositivity rate in this study was the same as that reported by Patitucci et al. [31] in 2000 in the southern part of the country. Unlike *T. gondii*, *N. caninum* appears to have a more consistent seroprevalence in the Southern Cone; specifically, in Chile, the two reported values are nearly identical despite corresponding to two distinct climate zones. Therefore, it would be warranted to increase the number of studies that account for climatic differences, thereby determining whether these differences have a real impact on the prevalence of *N. caninum*.

For *N. caninum*, the mixed-effects model showed singularity and a farm-level random-effect variance of zero, indicating no relevant residual clustering after accounting for fixed effects. Therefore, the final interpretation was based on a conventional multivariable logistic regression model, in which the use of straw as bedding (OR = 5.13) and the presence of cats (OR = 6.32) were considered risk factors. Other animals entering the food barn (OR = 0.02), the presence of farm dogs (OR = 0.12), the presence of farm dogs alongside unfamiliar dogs (OR = 0.16), and the use of the BCG vaccine (OR = 0.24) were associated with lower odds of seropositivity.

Regarding the use of straw as a bedding material and its implications for the occurrence of neosporosis, its influence is likely related to dogs’ preferences during defecation, or to its properties at certain temperatures or humidity levels that may favor the sporulation of *N. caninum* oocysts.

Regarding the presence of cats and their possible role in the onset of neosporosis, it is difficult to incorporate this into the parasite’s life cycle, but studies have already reported seropositivity across various feline species. Nazari et al. [57] determined, throughout a systematic review, an overall prevalence of 15% in felines. Another study on the occurrence of neosporosis in cattle in Namibia identified a moderate to high number of Feliformia that were positive for *N. caninum*, and they were 9.8 times more likely to be seropositive for *N. caninum* [58]. Thus, the presence of felines cohabiting with livestock and their influence on neosporosis should be studied further, as they may mask other important related factors.

Regarding the entry of animals into the shed, this variable included all animal types, and this result appears to mask other variables that may be protective against infection. For example, all farms had rodent control methods in place, but those where animals were more frequently sighted in the food storage area may have had stricter control measures. The characteristics of the warehouse building in each case also had to be taken into account, since, depending on materials, ventilation, and other structural factors, the temperature and humidity at which the food was stored could alter the conditions under which the food was stored, affecting oocyst sporulation.

Most studies report dogs as a risk factor. However, this factor is generally considered only in relation to the presence/contact of dogs with cattle [37,44,59] or to the number of canids on the property [60]. In the present study, our aim was to determine whether the role of dogs in the presentation of neosporosis depended solely on their presence, or whether their origin (own or unknown) also influenced it. Thus, it was found that in categories that included farm dogs, these were protective factors, unlike the category that included only dogs of unknown origin. Another study conducted in Taiwan examined this variable and reported that the presence of domestic dogs was negatively associated with *N. caninum* seropositivity, whereas the presence of stray dogs in the farm area was positively associated [61]. It is therefore plausible to consider that having dogs of one’s own, for reasons of territoriality, may limit the entry of other dogs of unknown origin onto the premises, thereby reducing exposure to potentially infected dogs.

The BCG vaccine has been an important tool for preventing tuberculosis in humans [62]. In Chile, several studies have demonstrated the relevance of its use in cattle [27,63,64], showing that it reduces tuberculosis incidence by 66.5% [27] and improves overall health [63]. However, BCG also has productive benefits, such as increasing milk production [27] and decreasing the somatic cell count and clinical mastitis [64]. The mechanisms responsible for these non-specific responses are not yet fully understood. However, they may involve modulation of the innate immune system through a process called trained immunity [65], defined as a long-lasting reprogramming that enhances the response of innate immune cells to subsequent infections, even those caused by unrelated pathogens, through changes at the epigenetic and gene-expression levels. These changes include enrichment of activating histone marks such as H3K4me3 and H3K27ac, reduction of repressive marks such as H3K9me3, and activation of glycolytic pathways linked to Akt/mTOR and HIF-1α signaling, which together may enhance secondary pro-inflammatory responses and favor M1-like macrophage polarization [66,67]. BCG-induced heterologous protection has also been described against other pathogens [19,20,21,22,28]. However, information on its effects against protozoan parasites remains limited. Notably, BCG-associated effects have been reported for *Babesia* and *Plasmodium* [23,24,25], two genera that, like *T. gondii* and *N. caninum*, belong to the phylum Apicomplexa. This shared phylogenetic background suggests that it is worthwhile to investigate whether heterologous immune mechanisms induced by BCG could also influence infections caused by *T. gondii* and *N. caninum*.

In this study, BCG vaccination was associated with reduced odds of *N. caninum* seropositivity in the multivariable model. However, given the observational nature of the study, these findings should be interpreted cautiously and cannot be considered evidence of a causal protective effect. Further experimental studies evaluating the potential influence of BCG vaccination on *N. caninum* infections in cattle would help clarify whether immunological mechanisms, such as trained immunity, could play a role in these associations. In this sense, these studies should address this question through prospective longitudinal or controlled experimental studies that include animals with known baseline serostatus, standardized BCG administration, measures of seroconversion for the agents in the study, repeated post-vaccination follow-up, and control for farm-level factors and co-interventions. One important point to consider is that the duration of this innate memory is not yet fully understood. Some studies in humans have reported a duration of at least 12 months [68]. Additionally, incorporating immunological measurements of trained immunity would be valuable for assessing whether a biologically plausible mechanism underlies any observed association.

This study provides relevant epidemiological data on *T. gondii* and *N. caninum* in intensive dairy cattle from central Chile and includes a comparatively broad sample of animals. In addition, although four farms did not complete the epidemiological survey and were therefore excluded from the risk factor analysis, comparisons between the full and restricted datasets showed no statistically significant differences in *T. gondii* seroprevalence, *N. caninum* seroprevalence, or the proportion of BCG-vaccinated animals. This suggests that the exclusion of farms with incomplete epidemiological information did not materially affect the main descriptive estimates. Nevertheless, some limitations should be considered when interpreting the findings. The limited number of farms included in the study may limit the generalizability of the results, and some bias from incomplete reporting cannot be entirely ruled out. In addition, because this is an observational cross-sectional study, the analysis may be affected by residual confounding and does not permit causal inference, particularly regarding the observed association between BCG vaccination and *N. caninum* seropositivity.

Finally, some epidemiological variables that could influence parasite transmission were not evaluated in detail. Future research should consider factors such as the behavior of dogs and cats across different areas of the farm, rather than only their presence or absence, as well as the proximity and characteristics of surrounding human settlements, which may affect the presence and movement of animals that contribute to parasite circulation. In addition, future studies should evaluate the long-term effects of BCG vaccination and its possible role in the epidemiology of these parasitic infections under longitudinal or experimental designs.

## 5. Conclusions

The seroprevalence of *T. gondii* and *N. caninum* in dairy cows from the central zone of Chile was 7.6% and 22.4%, respectively. For *T. gondii*, the factors associated with seropositivity were age groups of 3 to 4.5 years and over 4.5 years, as well as the presence of dogs in the pens. For *N. caninum*, straw bedding use and the presence of cats were associated with higher odds of seropositivity, whereas animal entry into the barn and the presence of farm dogs, either alone or together with unfamiliar dogs, were associated with lower odds of seropositivity. BCG vaccination was also associated with lower odds of *N. caninum* seropositivity; however, this finding should be interpreted cautiously, given the observational nature of the study, and should be evaluated in studies specifically designed to address this question. This is the first study to report *T. gondii* seropositivity in dairy cattle in Chile and the first to report *N. caninum* seropositivity in dairy cattle under an intensive production system in the central zone of Chile.

## Figures and Tables

**Table 1:** Seroprevalence rate for *Toxoplasma gondii* and *Neospora caninum* in cattle belonging to dairy farms located in Valparaíso (V), Metropolitana (MR), and O’Higgins (VI) regions of Chile.

		Percentage of Seropositivity (Frequency)	
Farm	Region	*T. gondii*	*N. caninum*
A *	MR	0.0% (0/44)	31.8% (14/44)
B *	MR	18.2% (6/33)	33.3% (11/33)
C	MR	10.8 (4/37)	8.1% (3/37)
D	MR	0.0% (0/7)	14.3% (1/7)
E *	MR	1.3% (1/79)	19% (15/79)
F *	MR	5.6% (2/36)	22.2% (8/36)
G *	MR	3.4% (3/87)	27.6% (24/87)
H	MR	10.0% (2/20)	35.0% (7/20)
I	MR	12.5% (5/40)	10.0% (4/40)
J	V	0.0% (0/42)	26.2% (11/42)
K	V	0.0% (0/21)	61.9% (13/21)
L	V	4.8% (1/21)	14.3% (3/21)
M	VI	2.5% (1/40)	15.0% (6/40)
N	VI	30.0% (18/60)	11.7% (7/60)
**Total**		**7.6% (43/567)**	**22.4% (127/567)**

* Indicates those farms that have been vaccinated with BCG.

**Table 2:** Variables associated with *T. gondii* infection in dairy cattle in Valparaíso, Metropolitana, and O’Higgins regions of Chile, according to multivariable mixed-effects logistic regression analysis.

Variables	Categories	*p*-Value	OR	Lower	Upper
Age (Years)	≤1.5	reference			
	1.5 < x ≤ 3	0.099	3.745	0.777	18.033
	3 < x ≤ 4.5	0.004 *	11.252	2.145	59.023
	>4.5	0.030 *	7.097	1.204	41.831
Dog presence in the pen	No	reference			
	Yes	0.032 *	6.075	1.164	31.690

* Statistically significant (*p* < 0.05). OR = Odds Ratio.

**Table 3:** Variables associated with *N. caninum* infection in cattle belonging to dairy farms in Valparaíso, Metropolitana, and O’Higgins regions of Chile, according to multivariate logistic regression analysis.

Variables	Categories	*p*-Value	OR	Lower	Upper
Animal entry into the barn	No	Reference			
	Yes	<0.001 *	0.018	0.003	0.116
Straw bedding use	No	Reference			
	Yes	0.001 *	5.133	1.978	13.318
Cat presence	No	Reference			
	Yes	0.023 *	6.323	1.292	30.937
Dog presence	No	Reference			
	Belong to the farm	0.001 *	0.116	0.033	0.410
	Unknown	0.194	0.615	0.296	1.280
	Belong to the farm and unknown	0.004 *	0.160	0.046	0.560
BCG use	No	Reference			
	Yes	0.004 *	0.239	0.091	0.631

* Statistically significant (*p* < 0.05). OR = Odds Ratio.

## Data Availability

The data presented in this study are available on request from the corresponding author.

## Supplementary Materials

The following supporting information can be downloaded at: https://www.mdpi.com/article/10.3390/ani16101456/s1, Table S1: Univariable analysis to determine risk factors for *Toxoplasma gondii* seropositivity in cattle from Valparaíso, Metropolitana, and O’Higgins regions of Chile; Table S2: Variables associated with *T. gondii* infection in dairy cattle in Valparaíso, Metropolitana, and O’Higgins regions of Chile, according to multivariable logistic regression analysis; Table S3: Univariable analysis to determine risk factors for *Neospora caninum* seropositivity in cattle from Valparaíso, Metropolitana, and O’Higgins regions of Chile.
