# Supplementary material for: Seropositivity and Risk Factors for Toxoplasma gondii and Neospora caninum in Intensive Dairy Cattle from Different Farms in Central Chile
Source: Animals (Basel). 2026 May 9;16(10):1456. doi: 10.3390/ani16101456 (PMC13203644; doi:10.3390/ani16101456)
Supplement: Supplementary file 1 [file animals-16-01456-s001.zip › Table S1. Univariable analysis to determine risk factors for Toxoplasma gondii seropositivity in cattle from Valparaíso, Metropolitana .pdf]

Table S1. Univariable analysis to determine risk factors for *Toxoplasma gondii* seropositivity in cattle from Valparaíso, Metropolitana and O'Higgins regions of Chile. *Statistically significant ($p < 0.05$). OR = Odds Ratio.

Variables	Categories	p-value	OR	95% CI	
				Lower	Upper
Square meters (m²) per animal	> 100	Reference			
	50 > x ≤ 100	0.510	0.703	0.246	2.008
	≤ 50	0.367	1.538	0.603	3.923
Seropositive for *Neospora caninum*	No	Reference			
	Yes	0.431	0.711	0.305	1.659
Other productions on the farm premises	No	Reference			
	Crops	0.050	0.290	0.084	0.998
	Cheese	0.177	1.750	0.776	3.947
	Meat	0.485	1.450	0.511	4.117
	Crops and meat	0.821	1.194	0.257	5.545
Age (years)	≤ 1.5	Reference			
	1.5 > x ≤ 3	0.189	2.355	0.738	10.447
	3 > x ≤ 4.5	0.004	6.426	2.054	28.305
	> 4.5	0.018	5.229	1.444	24.650
Location of the feeders	Only outside the pen	Reference			
	Inside and outside the pen	0.389	0.526	0.084	1.814
Other animals enter the food barn	No	Reference			
	Yes	0.777	1.140	0.461	2.818
Grazing/browsing	No	Reference			
	Yes	0.006	0.362	0.176	0.743
Number of bedding substrates used in the farm	One	Reference			
	Two	0.002	4.000	1.694	9.443
	Three or more	0.811	0.867	0.268	2.799
Travel time to the milking parlor (minutes)	≤ 5	Reference			
	5 > x ≤ 15	0.255	1.796	0.656	4.921
	> 15	0.000	5.017	2.182	11.536
Wood chip bedding	No	Reference			
	Yes	0.515	1.271	0.617	2.621
Straw bedding	No	Reference			
	Yes	0.000	0.301	0.155	0.584
Sand bedding	No	Reference			
	Yes	0.000	4.156	2.140	8.069
Compost bedding	No	Reference			

	Yes	0.119	0.554	0.264	1.164
Presence of cats	No	Reference			
	Yes	0.044	0.376	0.153	1.064
Waterer material	Cement	Reference			
	Plastic	0.002	25.241	3.360	189.606
	Metal	0.206	4.357	0.446	42.606
	Two or more of the above	0.009	15.250	1.949	119.346
Water source	Only from well	Reference			
	Well and potable water	0.058	2.518	0.969	6.541
	Well and natural sources	1.000	0.000	0.000	3318.109
Presence of dogs	No	Reference			
	Belong to the farm	0.229	2.270	0.596	8.645
	Unknown	0.037	3.773	1.084	13.133
	Belong to the farm and unknown	0.101	3.073	0.803	11.755
Dogs roam inside the pens	No	Reference			
	Yes	0.002	4.611	1.770	12.010
Dogs roam inside the milking parlor	No	Reference			
	Yes	0.746	1.152	0.489	2.715
Dogs roam inside the calf pens	No	Reference			
	Yes	0.468	0.698	0.264	1.844
Cats roam inside the pens	No	Reference			
	Yes	0.264	1.481	0.743	2.952
Cats roam inside the calf pens	No	Reference			
	Yes	0.068	0.459	0.199	1.059
Cats roam inside the milking parlor	No	Reference			
	Yes	0.264	1.481	0.743	2.952
BCG vaccine use	No	Reference			
	Yes	< 0.001	0.267	0.127	0.561
Region	Metropolitana	Reference			
	Valparaíso	1.000	0.001	0.000	2322.001
	O'Higgins	< 0.001	3.530	1.812	6.876
