# Supplementary material for: Seropositivity and Risk Factors for Toxoplasma gondii and Neospora caninum in Intensive Dairy Cattle from Different Farms in Central Chile
Source: Animals (Basel). 2026 May 9;16(10):1456. doi: 10.3390/ani16101456 (PMC13203644; doi:10.3390/ani16101456)
Supplement: Supplementary file 1 [file animals-16-01456-s001.zip › Table S2_Variables associated with T. gondii infection in dairy cattle in Valparaíso, Metropolitana, and O’iggins regions of Ch.pdf]

Table S2. Variables associated with *T. gondii* infection in dairy cattle in Valparaíso, Metropolitana, and O'Higgins regions of Chile, according to multivariable logistic regression analysis. *Statistically significant ($p < 0.05$). OR = Odds Ratio.

Variables	Categories	p-value	OR	Lower	Upper
Age (Years)	≤ 1.5	reference			
	1.5 < x ≤ 3	0.042*	5.424	1.064	27.652
	3 < x ≤ 4.5	0.001*	18.250	3.131	106.387
	> 4.5	0.018*	10.202	1.489	69.888
Straw bedding use	No	reference			
	Yes	0.037*	0.352	0.132	0.938
Sand bedding use	No	reference			
	Yes	0.011*	7.865	1.613	38.35
Compost bedding use	No	reference			
	Yes	0.222	2.939	0.522	16.564
Dog presence in the pen	No	reference			
	Yes	0.007*	9.886	1.884	51.881
Cat presence in the calf barns	No	reference			
	Yes	< 0.001*	0.074	0.021	0.267
BCG use	No	reference			
	Yes	0.066	4.574	0.902	23.187
