# Supplementary material for: Seropositivity and Risk Factors for Toxoplasma gondii and Neospora caninum in Intensive Dairy Cattle from Different Farms in Central Chile
Source: Animals (Basel). 2026 May 9;16(10):1456. doi: 10.3390/ani16101456 (PMC13203644; doi:10.3390/ani16101456)
Supplement: Supplementary file 1 [file animals-16-01456-s001.zip › Table S3. Univariable analysis to determine risk factors for Neospora caninum seropositivity in cattle from Valparaíso, Metropolitana a.pdf]

Table S3. Univariable analysis to determine risk factors for *Neospora caninum* seropositivity in cattle from Valparaíso, Metropolitana and O'Higgins regions of Chile. *Statistically significant ($p < 0.05$). OR = Odds Ratio.

Variables	Categories	p-value	OR	95% CI	
				Lower	Upper
Square meters (m^2) per animal	> 100	Reference			
	50 > x ≤ 100	0.027*	0.506	0.277	0.927
	≤ 50	0.038*	0.538	0.538	0.966
Seropositive for *T. gondii*	No	Reference			
	Yes	0.431	0.711	0.305	1.659
Other productions on the farm premises	No	Reference			
	Crops	0.002*	2.246	1.335	3.778
	Cheese	0.748	1.117	0.568	2.199
	Meat	0.184	0.479	0.162	1.419
	Crops and meat	0.188	1.989	0.714	5.538
Age (years)	≤ 1.5	Reference			
	1.5 > x ≤ 3	0.535	0.831	0.464	1.490
	3 > x ≤ 4.5	0.259	1.441	0.764	2.717
	> 4.5	0.965	0.983	0.455	2.125
Location of the feeders	Only outside the pen	Reference			
	Inside and outside the pen	< 0.001*	3.598	1.850	6.996
Other animals enter the food barn	No	Reference			
	Yes	0.019*	0.523	0.305	0.898
Grazing/browsing	No	Reference			
	Yes	0.720	0.923	0.595	1.432
Number of bedding substrates used in the farm	One	Reference			
	Two	0.001*	0.369	0.205	0.664
	Three or more	0.141	1.472	0.880	2.461
Travel time to the milking parlor (minutes)	≤ 5	Reference			
	5 > x ≤ 15	0.001*	2.328	1.403	3.862
	> 15	0.039*	0.527	0.286	0.969
Wood chip bedding	No	Reference			
	Yes	0.331	1.270	0.784	2.059
Straw bedding	No	Reference			
	Yes	0.077	1.573	0.953	2.598
Sand bedding	No	Reference			
	Yes	0.396	0.798	0.474	1.343
Compost bedding	No	Reference			

	Yes	0.029*	1.644	1.052	2.567
Presence of cats	No	Reference			
	Yes	0.100	0.526	0.245	1.130
Waterer material	Cement	Reference			
	Plastic	0.016*	0.448	0.232	0.863
	Metal	0.510	1.235	0.659	2.314
	Two or more of the above	0.233	1.426	0.795	2.557
Water source	Only from well	Reference			
	Well and potable water	0.049*	2.162	1.001	4.667
	Well and natural sources	< 0.001*	6.706	2.687	16.732
Presence of dogs	No	Reference			
	Belong to the farm	0.085	0.557	0.286	1.083
	Unknown	0.316	0.735	0.403	1.341
	Belong to the farm and unknown	0.727	0.888	0.456	1.729
Dogs roam inside the pens	No	Reference			
	Yes	< 0.001*	0.382	0.244	0.597
Dogs roam inside the milking parlor	No	Reference			
	Yes	0.804	1.078	0.595	1.954
Dogs roam inside the calf pens	No	Reference			
	Yes	0.018*	0.412	0.198	0.857
Cats roam inside the pens	No	Reference			
	Yes	0.177	0.737	0.474	1.147
Cats roam inside the calf pens	No	Reference			
	Yes	0.052	0.536	0.286	1.005
Cats roam inside the milking parlor	No	Reference			
	Yes	0.177	0.737	0.474	1.147
BCG use	No	Reference			
	Yes	0.033*	1.632	1.041	2.557
Region	Metropolitana	Reference			
	Valparaíso	< 0.001*	5.487	2.194	13.723
	O'Higgins	0.035*	0.505	0.267	0.953
